# Neutralizing Antibody Responses to *Chlamydia trachomatis* in Women and Associations With Chlamydia Outcomes

**DOI:** 10.1093/infdis/jiae519

**Published:** 2024-10-22

**Authors:** Hong Yu, William M Geisler, Chuanbin Dai, Kanupriya Gupta, Gary Cutter, Robert C Brunham

**Affiliations:** British Columbia Centre for Disease Control, University of British Columbia, Vancouver, Canada; Department of Medicine, University of Alabama at Birmingham; British Columbia Centre for Disease Control, University of British Columbia, Vancouver, Canada; Department of Medicine, University of Alabama at Birmingham; Department of Biostatistics, University of Alabama at Birmingham; British Columbia Centre for Disease Control, University of British Columbia, Vancouver, Canada

**Keywords:** chlamydia infection, clinical outcomes, neutralizing antibody, serology, women

## Abstract

We assessed neutralizing antibody responses in a well-characterized cohort of 60 women with different *Chlamydia trachomatis* infection outcomes noted at a treatment visit and 3-month follow-up. We found varying rates of neutralization (inhibition of *C trachomatis*) in sera at different dilution levels and varying neutralizing antibody titers across outcomes. Median neutralization rates were significantly higher in sera at high dilutions (1:320–1:1280) from women with spontaneous resolution vs persisting infection before treatment (all *P* < .05). These findings suggest that neutralizing antibody responses may contribute to protective immunity against chlamydia.

## Teaser

This study examined *C trachomatis* neutralizing antibody responses in a well-characterized cohort of 60 women with different chlamydia outcomes. The findings suggest that neutralizing antibody responses may contribute to protective immunity against chlamydia in humans.

*Chlamydia trachomatis* infection (ie, chlamydia) is the leading cause of sexually transmitted bacterial infection, posing risks for complications in women, such as pelvic inflammatory disease, ectopic pregnancy, and infertility. Despite collaborative public health efforts focused on case identification, treatment, and contact tracing, chlamydia rates have increased almost every year [1], perhaps in part due to blunting of immunity development by early treatment [2]. Consequently, a vaccine is likely the most effective means for chlamydia control.

A critical aspect of chlamydia vaccine development is determining immune correlates of protection against chlamydia in humans. Murine chlamydia studies have shown that CD4+ T-helper type 1 responses, specifically interferon γ (IFN-γ) mediated, are essential for initial infection clearance and pathology prevention; however, T-helper type 1 and antibody responses contribute to protection against reinfection [3]. Given that murine data suggest that antibody responses are important in protection against reinfection, it is crucial to determine the role of antibody responses, especially functional responses such as opsonophagocytic and neutralizing antibodies, in protecting humans against chlamydia.

In a recent study, we utilized a flow cytometry–based assay using neutrophil-like cells to quantify antibody-mediated phagocytosis of *C trachomatis* elementary bodies (EBs). We found that while antibody-mediated phagocytosis was detected in most women treated for chlamydia and opsonophagocytic antibodies correlated with immunoglobulin G1 antibodies against multiple *C trachomatis* proteins, opsonophagocytic antibody responses were not associated with clinical correlates of protection [4].

Research on neutralizing antibodies against *C trachomatis* has been reported in sparse human studies [5]. However, there have been no reports on the association of neutralizing antibody responses in relation to chlamydia outcomes that may be clinical correlates of protection. In this study, we examined *C trachomatis* neutralizing antibody responses in a well-characterized cohort of 60 women with different chlamydia outcomes, including 2 outcomes likely associated with protective immunity: spontaneous resolution of infection and absence of reinfection posttreatment [6, 7]. Our aim was to determine if *C trachomatis* neutralizing antibody responses were associated with clinical correlates of protection.

## METHODS

### Study Population

We evaluated serum neutralizing antibody responses in 60 women from a cohort returning for treatment of a positive *C trachomatis* screening test result (enrollment visit; V1) and at a 3-month follow-up visit (V2), as described elsewhere [4, 6]; the average time between a positive test result and V1 was 10.2 days. Briefly, women were categorized into 3 clinical outcome groups based on *C trachomatis* nucleic acid amplification test (NAAT) results at V1 and V2.

Spontaneous resolution: spontaneous resolution of chlamydia before treatment (V1 NAAT negative) and no reinfection at follow-up (V2 NAAT negative)

Persisting infection without reinfection: persistent infection at V1 (V1 NAAT positive) but no reinfection at V2 (V2 NAAT negative)

Persisting infection with reinfection: persistent infection at V1 (V1 NAAT positive) and reinfection at V2 (V2 NAAT positive).

We also evaluated antibody responses in serum from 20 women who were *C trachomatis* naive (ie, negative controls with no known history of chlamydia, a negative genital *C trachomatis* NAAT result, and *C trachomatis* seronegative) from a cohort described elsewhere [8]. The study was approved by the University of Alabama at Birmingham Institutional Review Board.

### *C trachomatis* Neutralizing Assay

Sera were heat inactivated at 56 °C for 30 minutes before neutralization testing. Methodology has been reported [9]. Briefly, Hela 229 cells were seeded in 96-well plates and grown overnight until 70%–80% confluence. Of 10 000 inclusion-forming units of *C trachomatis* EB serovar D diluted in sucrose-phosphate-glutamate buffer, 100 μL was added to 100 μL of 2-fold serially diluted serum from 1:10 to 1:1280 and incubated for 30 minutes at 37 °C. Samples incubated with EB but without serum were used as negative control. Then, 50 μL of the mixture of serum and EBs (2500 inclusion-forming units) was added to each well. The plate was spun at 3000 rpm (room temperature) for 30 minutes, followed by incubation at 37 °C for 1.5 hours. Samples were removed and replaced with fresh medium (Eagle's Minimum Essential Medium supplemented with 10% fetal calf serum) containing cycloheximide (1 μg/mL). Cells were incubated for 48 hours and fixed for *C trachomatis* inclusions, which were visualized by staining with anti-EB mouse antibody, followed by horseradish peroxidase–conjugated donkey anti-mouse IgG (Jackson ImmunoResearch) and a 3,3-diaminobenzidine substrate (Thermo Scientific).

Inclusions were visualized with a Nikon ECLIPSE Ti. Nine images per well were captured at 20×, and inclusions were enumerated by NIS-Elements Imaging Software (Supplementary Figure 1). Neutralization rates (percentage inhibition of infection) were calculated as follows: inhibition = [1 – (inclusion count of samples / inclusion count of negative control)] × 100%. Half-maximal inhibitory concentration (IC50) values—the serum dilution resulting in a 50% reduction in the number of *C trachomatis* inclusions grown in each well—were calculated with Prism version 9.5 (GraphPad) and nonlinear regression analysis.

### Statistical Analyses

Data were analyzed with JMP PRO version 16.0.0 (SAS Institute Inc). Differences in neutralizing antibody responses among chlamydia outcome groups and between women with chlamydia outcomes and those who were *C trachomatis* naive (control) were analyzed with the Mann-Whitney test. Differences in neutralizing antibody responses between V1 and V2 in women with chlamydia outcomes were analyzed by the paired *t* test. *P* < .05 was considered significant.

## RESULTS

### Neutralizing Antibody Responses in Women With Chlamydia Outcomes and Women Who Are *C trachomatis* Naive

**Figure 1.**
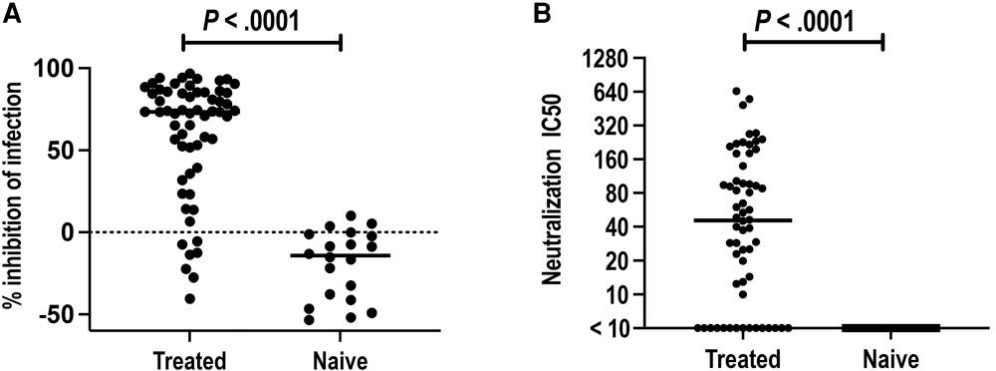
*Chlamydia trachomatis* neutralizing antibody responses in women presenting for treatment of chlamydia and in women who were *C trachomatis* naive. A comparison was made between neutralizing antibodies against *C trachomatis* serovar D elementary bodies (EBs) in 60 serum samples at the enrollment visit from 60 women presenting for treatment of chlamydia (treated) and in 20 serum samples from 20 women who were *C trachomatis* naive (naive). *A*, Percentage inhibition of infection (neutralization rate) in serum samples with a 1:10 dilution. *B*, Neutralization half-maximal inhibitory concentration (IC50) of serum samples. *A* and *B*, Percentage inhibition of infection and neutralization IC50 were significantly higher in women treated for chlamydia vs women who were *C trachomatis* naive. Statistical significance was analyzed by Mann-Whitney test. Horizontal bar represents the median.

The median neutralization rate of *C trachomatis* in the 60 V1 serum samples in women returning for treatment of a *C trachomatis*–positive NAAT result was significantly higher than that in the 20 *C trachomatis*–naive sera at a 1:10 serum dilution (73.35% vs −14.15%, *P* < .0001; Figure 1*A*). This significant difference persisted even when sera were diluted to 1:320 (Supplementary Figure 2).

Figure 1 *B* illustrates the median neutralization IC50 in the same serum samples. Sera from women treated for chlamydia displayed varying levels of neutralizing antibody titers (IC50) against *C trachomatis* EBs, with a median IC50 of 46.7 (range, <10 to 648.4) and with 25% exhibiting an IC50 <10. As expected, the IC50 of all *C trachomatis*–naive sera was <10.

### Neutralizing Antibody Responses Across Baseline and Follow-up Visits in Women With Chlamydia Outcomes

**Figure 2.**
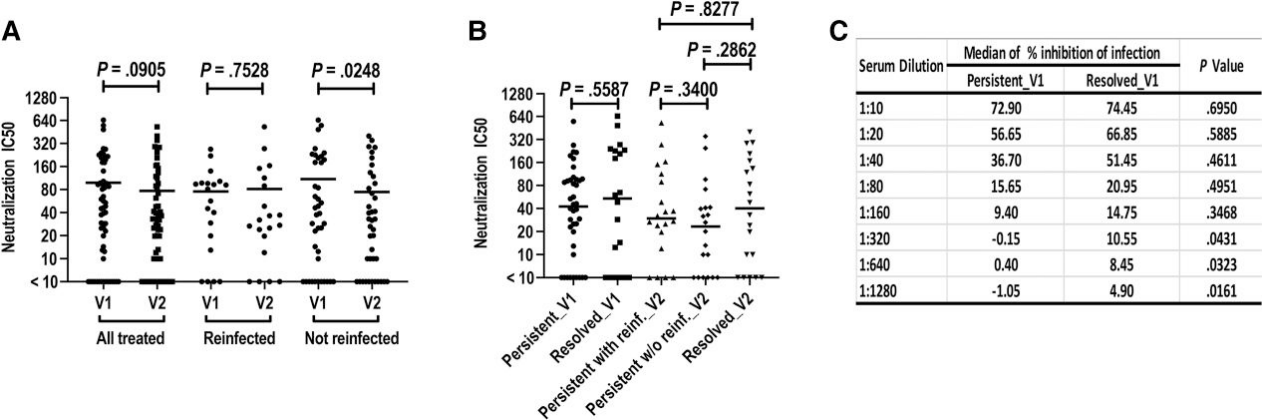
*Chlamydia trachomatis* neutralizing antibody titers across enrollment and 3-month follow-up visits and association of neutralizing antibody responses with different chlamydia outcomes. *A*, Mean neutralization half-maximal inhibitory concentration (IC50) values at an enrollment visit (V1) vs 3-month follow-up visit (V2) in women: all treated (N = 60), reinfected (n = 20), and not reinfected (n = 40). *B*, Median neutralization IC50 values of serum from groups with different chlamydia outcomes at V1 and V2: spontaneous resolved (n = 20) vs persisting infection (n = 40) at V1 and presence of reinfection (n = 20) vs no reinfection (n = 20) at V2 stratified by persistent infection status at V1. Resolved_V1 refers to the spontaneous resolution of chlamydia before treatment at V1, and all women in this group had no reinfection at V2 (Resolved_V2). *C*, Median percentage inhibition of infection of *C trachomatis* serovar D elementary bodies in V1 serum samples with different dilutions. *A*, There was a trend toward a decline in mean neutralization IC50 from V1 to V2, which was driven by a significant decline in those without reinfection. *B*, Median neutralization IC50 was not significantly associated with chlamydia outcomes. *C*, Medians of percentage inhibition of infection were significantly higher in those with spontaneous resolution vs persisting infection at V1 at serum dilutions of 1:320 or higher but not at lower serum dilutions. Statistical significance was analyzed by (*A*) a paired *t* test and (*B* and *C*) a Mann-Whitney test.

Neutralizing antibody titers somewhat declined between V1 and V2, with a higher IC50 at V1 vs V2 (mean ± SD, 97.0 ± 135.0 vs 75.6 ± 112.2; *P* = .0905). However, when stratified by reinfection status at follow-up, those without reinfection had a significant decline in titers (*P* = .0248) while those with reinfection did not (Figure 2*A*); significant or trending differences in neutralization rates at serum dilutions of 1:10 through 1:320 between the visits validated this observation (Supplementary Figure 3).

### Neutralizing Antibody Responses in Relation to Chlamydia Outcomes

Among the 60 women with chlamydia outcomes, we evaluated neutralizing antibody responses in 20 per group (spontaneous resolution, persisting infection without reinfection, and persisting infection with reinfection) at V1 and V2 as illustrated in Figure 2*B*. No significant difference was found in median neutralizing antibody titers (IC50) across chlamydia outcomes at both visits. However, the possible contribution of neutralizing antibody responses to clinical protection was supported by finding significantly higher median neutralization rates in serum at higher dilutions (1:320, 1:640, and 1:1280) in the spontaneous resolution group vs the persisting infection groups at V1 (all *P* < .05; Figure 2*C*, Supplementary Figure 4).

## DISCUSSION

Our previous research in women who spontaneously resolve chlamydia suggested that natural immunity to *C trachomatis* confers some degree of protective immunity against reinfection [7]. Animal research indicates that antibodies contribute significantly to clearing chlamydia upon secondary infection [3]. Antibodies could function to prevent infection of epithelial cells and facilitate clearance of chlamydia through various mechanisms, including extracellular or intracellular neutralization, Fc-mediated processes such as antibody-dependent phagocytosis, and enhancement of T-cell responses such as IFN-γ production [10].

Our current study revealed that women presenting for treatment of chlamydia exhibited varying serum neutralizing antibody titers (IC50) and rates of neutralization of *C trachomatis* in sera. While these neutralizing antibody responses were not associated with clinical correlates of protection at lower serum dilutions, we found that at higher serum dilutions, women with spontaneous chlamydia resolution had significantly higher neutralization rates. Given that spontaneous chlamydia resolution may be a clinical correlate of immune protection, these findings suggest that neutralizing antibody responses may contribute to protective immunity to chlamydia in humans. Our finding that neutralizing antibody responses somewhat declined over a 3-month follow-up period suggests that they may be short-lived, but longer follow-up would be needed to assess this.

Murine studies have shown that CD4 T-cell–secreted IFN-γ is a key component of a protective antibody response in *Chlamydia* infection: IFN-γ appears to be necessary for activating effector cells that function in antibody-mediated immunity to reinfection [10]. For instance, primary peritoneal neutrophils stimulated with IFN-γ, as well as nonstimulated primary peritoneal neutrophils treated with immune serum, were unable to decrease *Chlamydia* load in a phagocytic killing assay. However, IFN-γ–stimulated primary peritoneal neutrophils treated with immune serum resulted in an almost 100-fold decrease [11]. Our previous finding was that women with spontaneous chlamydia resolution had a higher magnitude of IFN-γ against selected *C trachomatis* proteins [12]. In our current study, we found that spontaneous resolution is associated with stronger neutralizing antibody responses. As such, both findings support the murine data on the potential importance of T-cell and antibody responses in protective immunity. Our recent study [4] found no association of antibody-mediated phagocytosis with clinical protection, which suggests that neutralizing antibodies may be more important than opsonization in protective immunity to chlamydia in humans. Thus, in women, it is likely that *C trachomatis* neutralizing antibody-mediated protection is largely dependent on IFN-γ produced by CD4 T cells.

Phase 1 clinical trials of the first human *C trachomatis* vaccine, CTH522-CAF01—which contains a recombinant version of *C trachomatis* major outer membrane protein (CTH522) engineered to stimulate broadly neutralizing antibodies against urogenital and ocular *C trachomatis* serovars, as well as a novel adjuvant (CAF01)—showed that vaccination with CTH522-CAF01 induced elevated levels of neutralizing antibodies and CD4 IFN-γ [13, 14]. In fact, the median neutralizing antibody titer against serovar D was up to roughly 1000, much higher than the median and mean titers of neutralizing antibodies against serovar D in our study (45.7 and 97.0, respectively). This finding is likely explained in part by vaccine recipients not having current chlamydia and receiving multiple doses (challenges) of a vaccine concentrated with a purified immunogenic *C trachomatis* antigen and an adjuvant, as opposed to our cohort, which consisted of those naturally infected (recently or currently) with whole *C trachomatis* bacteria. Additionally, differences in neutralization assays—such as the vaccine studies with HaK cells without centrifugation vs our study with HeLa cells with centrifugation to assist infection—may explain the lower neutralization titers that we observed. Rosenkrands et al [15] showed that a key difference in MOMP VD4–specific antibodies from infection vs vaccination was that infection triggers a heterogeneous VD4-specific antibody response, while CTH522-CAF01 vaccination induces a uniform VD4 response with 4-times higher neutralizing titers. Although the vaccine studies showed higher neutralizing antibodies, they have not evaluated the correlation of these responses with protective immunity, which will be important to verify our findings suggesting that neutralizing antibody responses contribute to protective immunity. Our finding that neutralizing antibodies were higher in those with recent reinfection supports the notion that repeat *C trachomatis* exposures boost neutralizing antibody titers and may be important in maintaining antibody-mediated adaptive immunity in concert with the protective role of IFN-γ.

It is important to acknowledge that a potential limitation in our study is that we measured neutralizing antibody responses in serum against only *C trachomatis* serovar D, a highly prevalent serovar, which may not accurately reflect the significance of these responses in women infected with other serovars. Other limitations include our sample size, limited duration of follow-up, and not evaluating mucosal antibody responses (a future interest).

In conclusion, our study demonstrated that women mount neutralizing antibody responses to *C trachomatis* and that, at higher serum dilutions, the antibody responses were associated with a clinical correlate of protection, spontaneous chlamydia resolution prior to treatment, which suggests that neutralizing antibody responses may contribute to protective immunity against chlamydia in humans. Clinical trials of *C trachomatis* vaccines that evaluate the impact of vaccine-induced immunity on clinical protection should shed light on the importance of *C trachomatis*–specific functional antibody responses in protective immunity and their duration.

## Supplementary Data

Supplementary materials are available at *The Journal of Infectious Diseases* online (http://jid.oxfordjournals.org/). Supplementary materials consist of data provided by the author that are published to benefit the reader. The posted materials are not copyedited. The contents of all supplementary data are the sole responsibility of the authors. Questions or messages regarding errors should be addressed to the author.

## Supplementary Material

jiae519_Supplementary_Data

## Notes

***Acknowledgments.*** We thank the research personnel and study participants from the Sexual Health Clinic at the Jefferson County Department of Health for their valuable contributions.

***Author contributions.*** H. Y. developed and performed assays, evaluated results, interpreted the data, and wrote the manuscript. W. M. G. and R. C. B. conceptualized the study, evaluated results, interpreted the data, and wrote the manuscript. W. M. G. also provided the clinical data. C. D. developed and performed assays and evaluated results. K. G. processed and shipped sera for the assays, interpreted the data, and edited the manuscript. G. C. conducted statistical analyses and edited the manuscript. All authors contributed to the article and approved the submitted version.

***Financial support*.** This work was supported by the National Institute of Allergy and Infectious Diseases of the National Institutes of Health (R01AI093692 and R01AI148359 to W. M. G.).
