# Supplementary material for: Neutralizing Antibody Responses to Chlamydia trachomatis in Women and Associations With Chlamydia Outcomes
Source: J Infect Dis. 2024 Oct 22;231(3):e531–5. doi: 10.1093/infdis/jiae519 (PMC11911780; doi:10.1093/infdis/jiae519)
Supplement: jiae519_Supplementary_Data [file jiae519_supplementary_data.pptx]

## Slide 1
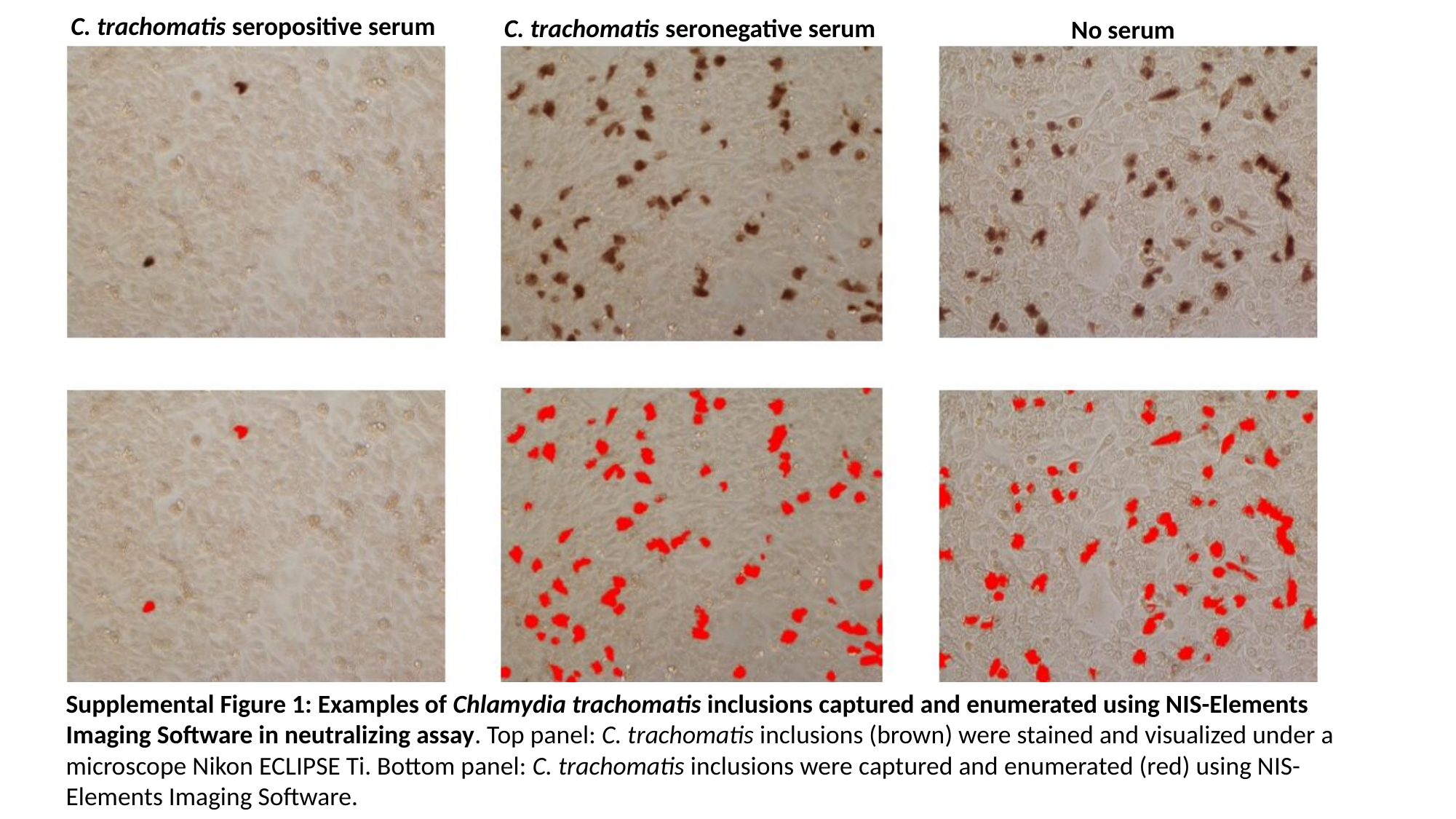

C. trachomatis seropositive serum
C. trachomatis seronegative serum
No serum
Supplemental Figure 1: Examples of Chlamydia trachomatis inclusions captured and enumerated using NIS-Elements Imaging Software in neutralizing assay. Top panel: C. trachomatis inclusions (brown) were stained and visualized under a microscope Nikon ECLIPSE Ti. Bottom panel: C. trachomatis inclusions were captured and enumerated (red) using NIS-Elements Imaging Software.

## Slide 2
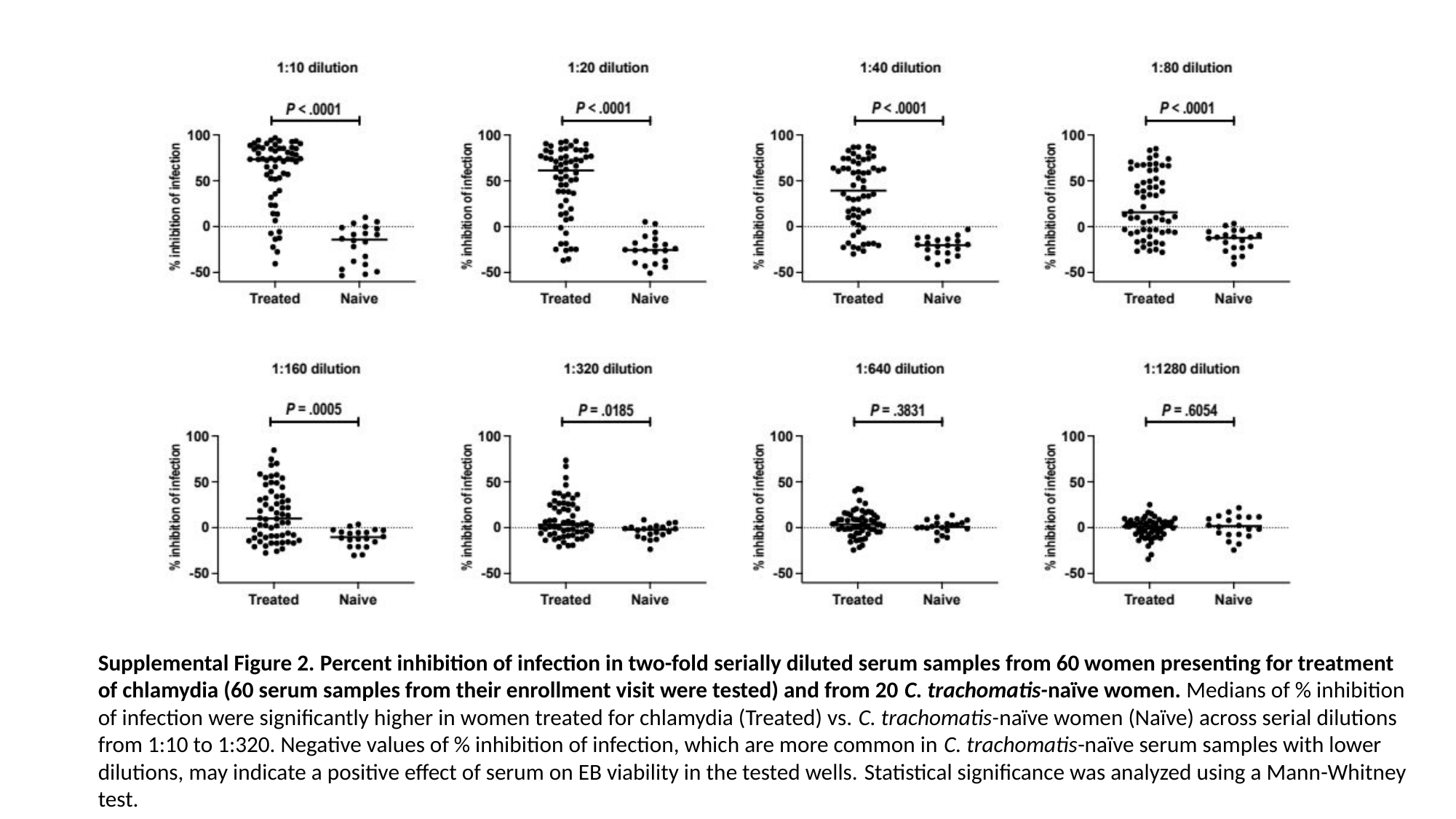

Supplemental Figure 2. Percent inhibition of infection in two-fold serially diluted serum samples from 60 women presenting for treatment of chlamydia (60 serum samples from their enrollment visit were tested) and from 20 C. trachomatis-naïve women. Medians of % inhibition of infection were significantly higher in women treated for chlamydia (Treated) vs. C. trachomatis-naïve women (Naïve) across serial dilutions from 1:10 to 1:320. Negative values of % inhibition of infection, which are more common in C. trachomatis-naïve serum samples with lower dilutions, may indicate a positive effect of serum on EB viability in the tested wells. Statistical significance was analyzed using a Mann-Whitney test.

## Slide 3
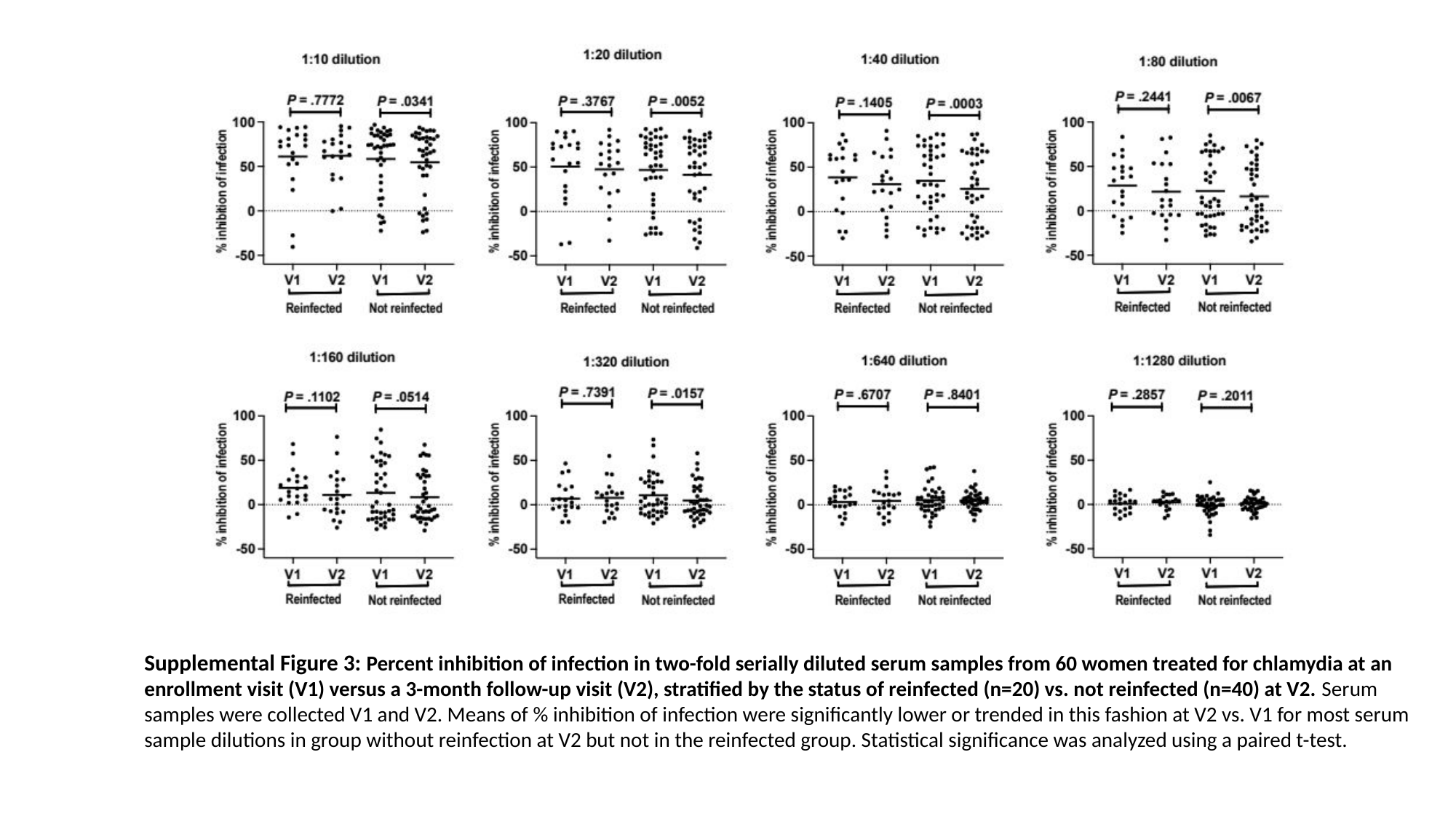

Supplemental Figure 3: Percent inhibition of infection in two-fold serially diluted serum samples from 60 women treated for chlamydia at an enrollment visit (V1) versus a 3-month follow-up visit (V2), stratified by the status of reinfected (n=20) vs. not reinfected (n=40) at V2. Serum samples were collected V1 and V2. Means of % inhibition of infection were significantly lower or trended in this fashion at V2 vs. V1 for most serum sample dilutions in group without reinfection at V2 but not in the reinfected group. Statistical significance was analyzed using a paired t-test.

## Slide 4
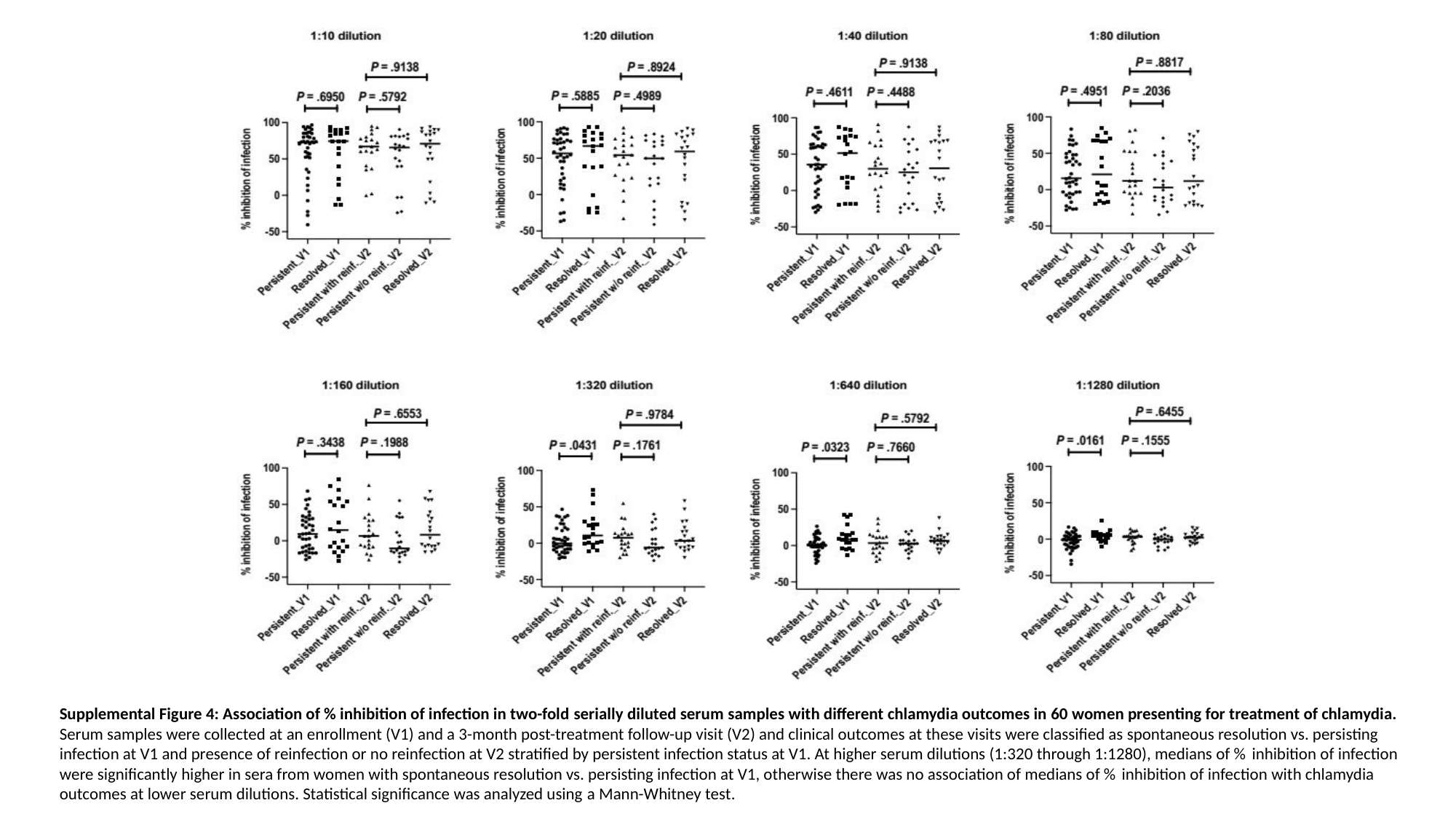

Supplemental Figure 4: Association of % inhibition of infection in two-fold serially diluted serum samples with different chlamydia outcomes in 60 women presenting for treatment of chlamydia. Serum samples were collected at an enrollment (V1) and a 3-month post-treatment follow-up visit (V2) and clinical outcomes at these visits were classified as spontaneous resolution vs. persisting infection at V1 and presence of reinfection or no reinfection at V2 stratified by persistent infection status at V1. At higher serum dilutions (1:320 through 1:1280), medians of % inhibition of infection were significantly higher in sera from women with spontaneous resolution vs. persisting infection at V1, otherwise there was no association of medians of % inhibition of infection with chlamydia outcomes at lower serum dilutions. Statistical significance was analyzed using a Mann-Whitney test.
